# Rotaviruses and Rotavirus Vaccines: Special Issue Editorial

**DOI:** 10.3390/v16111665

**Published:** 2024-10-24

**Authors:** John T. Patton, Ulrich Desselberger

**Affiliations:** 1 Department of Biology, Indiana University, 212 S Hawthorne Drive, Simon Hall 011, Bloomington, IN 47405, USA; 2 Department of Medicine, University of Cambridge, Addenbrooke’s Hospital, Hills Road, Cambridge CB2 0QQ, UK; ud207@medschl.cam.ac.uk

Species A rotaviruses (RVA) are a major cause of acute gastroenteritis in infants and young children and in the young of various mammalian and avian species [1,2,3]. Since 2006, several live attenuated RV vaccines have been licensed and are now components of childhood immunization programs in >100 countries worldwide [4,5]. However, vaccine efficacy/effectiveness (VE) varies in different countries [6,7]. As summarized elsewhere, much has been learned about many aspects of rotavirus biology, including its entry and assembly pathways, mechanism of genome replication, the structure and function of its proteins, and its basis of pathogenesis [2,3]. The study of rotavirus biology was significantly advanced about 6 years ago when reliable, plasmid-only-based reverse genetics (RG) systems for species A RVs (RVA) were established [8,9,10]. In this Special Issue of *Viruses* on *Rotaviruses and Rotavirus Vaccines*, novel data on the use of RVA RG, RVA replication, RVA molecular epidemiology, and RVA next-generation (NG) RV candidate vaccine developments are presented in 21 contributions and assessed in the larger context.

RG procedures have been used to rescue recombinant (r)RVAs of simian, human, murine, porcine, bovine and avian origin, as well as reassortants thereof, as summarized in Table 1 [8,9,11,12,13,14,15,16,17,18,19,20,21,22,23,24,25,26,27]. Murine RVs (MuRVs) replicate well in their natural host and are therefore an attractive animal model system. Using RG procedures, Kawagishi et al. [18] succeeded in rescuing recombinant RVs containing all 11 RNA segments of murine origin (rMuRV). Oligo-reassortants of rMuRV containing human VP7 and VP4 genes were shown to replicate efficiently in mice and to elicit a robust antibody response to human rotavirus antigens, whilst the control chimeric murine rotavirus did not [18,19]. Fukuda et al. [16] generated an rRV from the RV vaccine strain RIX4414 (Rotarix^®^) which was live attenuated in a mouse model. The porcine RV OSU strain (G5P[7]) was completely rescued as an rRVA and also engineered to express the fluorescent reporter protein UnaG [21]. Recently, an rRV of the Chinese Lanzhou lamb RV LLR was established [23]. These constructs can be used in the gnotobiotic (gn) piglet model ([28,29], see below) and will permit the development of urgently needed, next-generation porcine RV vaccines. Fluorescing rRVs can be used in large-scale neutralization assays to assess antibody responses in vaccinees ([8,30], see below). These developments in RVA RG research should be seen in the larger context of highly original recent work. Using a virus-like, codon-modified transgene, it was possible to generate a stable dsRNA virus as a potential vector [31]. An rRVA with a glycosylation-defective NSP4 gene showed attenuated replication in cultured cells and also less pathogenicity than its corresponding wildtype virus in a mouse model [32]. Furthermore, the combination of the structure–function-based molecular data of RVA proteins and RG techniques permitted the creation of an rRV which was infectious for only a single round of replication and thus an interesting candidate for a next-generation RV vaccine ([33], see below).

Regarding RV replication, the introduction of a point mutation into the VP4 gene of a human/simian triple reassortant rRVA strain led to increased replication in cell cultures; however, the effect was highly VP4 genotype-specific [34]. Human RVA clinical isolates initially replicate poorly in continuous cell lines, e.g., MA104 cells, but can be adapted to higher replication rates upon serial cell passage. This was observed with human RV isolates of five different genotype constellations upon passage: Illumina NG sequencing of the passaged viruses demonstrated the emergence of multiple point mutations, which, however, were partially conserved for the VP4 genes throughout genotypes [35].

In search of antivirals specifically blocking cellular compounds, a small molecule, ML241 (an ATPase inhibitor) was identified which blocked RVA replication at an IC_50_ of 22.5 uM in vitro and in vivo (suckling mouse model) via inhibition of MAPK signaling, leading to activation of the NF-kB pathway [36].

Germfree gnotobiotic (gn) piglets were colonized with apathogenic-bacteria-expressing histo-blood group antigens (HBGA^+^) that are well recognized in regard to binding to RVAs. Upon RV infection, the HBGA^+^ colonized animals showed reduced disease severity and virus shedding compared to the HBGA^-^ animals. This finding can lead to further identification of bacteria with substantial probiotic activity [37].

The formation and kinetics of viroplasms during RVA infection has been shown to depend on interactions with cytoskeleton proteins (microtubules, kinesin E5, intermediate filaments, myosin a.o.) [38]. In this context, the interaction of RV NSP5, a viroplasm building block, with the tailless complex polypeptide I ring complex (TRiC), a cellular chaperonin involved in the folding of cellular proteins, was discovered [39]. Inhibition of TRiC expression was shown to reduce RV replication. There is also the interaction of various components of cellular lipid metabolism with viral proteins not only during the replication of RVAs but also with the proteins of many other RNA viruses, a discovery with the potential to help in developing broadly active antivirals [40].

RV-encoded NSP2 interacts with NSP5 by liquid–liquid phase separation to form the biomolecular condensates of viroplasms [41]. NSP2 and NSP5 have enzymatic activities and are involved in RNA-RNA interactions during assortment in early RV morphogenesis; in this, NSP2 acts as a viral chaperone for the viral RNAs [42]. There are still questions about how the enzymatic activities of the viroplasm proteins are correlated with their functions during RVA replication and how assortment of the 11 pregenomic ss(+)RNA segments is controlled [42]. The groups of E Gaunt and P Digard joined forces to analyze the molecular details of the transcription/replication machinery of species A rotaviruses [43]. They constructed a mini-replicon assay which can serve as an important tool in determining the functional correlates of the viral RNA-dependent RNA polymerase (RdRp; [43]).

Hellysaz and Hagbom [44] reviewed what is known about the correlation of rotavirus disease symptoms with pathophysiological data, emphasizing CNS responses to the enteric infection.

The co-expression of RVA NSP5 with either NSP2 or VP2 in uninfected cells was found to lead to the formation of viroplasm-like structures (VLSs) several decades ago [45]. The knowledge gap regarding this structure–function relationship in non-RVA viroplasms is due to the lack of specific antibodies and suitable cell culture systems. In a recent study, the ability of the NSP5 and NSP2 of non-RVA species to form VLSs was explored [46]. While co-expression of these two proteins led to globular VLSs in RV species A, B, D, F, G, and I, in RVC, filamentous VLSs were formed. Remarkably, the co-expression of the NSP5 and NSP2 of the RV species H and J did not result in VLS formation. Interestingly, interspecies VLSs were formed between the relevant components of closely related RV species B with G and D with F [46]. This innovative data set is considered to form the basis of numerous follow-up experiments.

Rapid progress in nucleic acid sequencing techniques allows for more detailed analyses of the molecular epidemiology of RVAs. Species A, B, and C RV isolates were obtained from a pig farm in South Africa. Among thr 12 RVA isolates of G5 genotype, reassortment with three different P genotype genes (encoding P[6], P[13], and P[23]) were detected on an otherwise unchanged genetic background [47]. A G5P[23] porcine RV isolated from a pig farm in China carried several genes closely related to the cogent (analogous) genes of human RV isolates, strongly suggesting that it had emerged from a human–porcine RV reassortment event [48]. In China, >20,000 RVA strains were isolated from pigs during 2022, with G9P[23]I5 virus strains being the most prevalent [49]. Some of the porcine isolates were closely related genetically to human RVA strains, strongly suggesting that zoonotic transmissions occurred [49]. Similar results were recorded in other areas of the world [50]. G9P[6] and G9P[4] strains were isolated from children in Mozambique after the introduction of RVA vaccines [51].

RG procedures were used to insert a reporter gene (encoding green fluorescent protein, GFP, or others) downstream of NSP1 (or NSP5), separated by the 2A sequence from the viral gene. These rRV were used in micro-neutralization assays, permitting screening of large numbers of sera [30]. The procedure also demonstrated the presence of pre-existing immunity to rotaviruses in the sera of humans and various animal species (rhesus monkey, rabbit, mouse, guinea pig, and cotton rat).

Existing RV vaccines are live attenuated, can reassort with co-circulating RV wildtype strains, and may revert to virulence. Therefore, present efforts for next-generation RV vaccines are focused on antigens of non-replicating RVs [33,52,53]. In the search for a broad-spectrum, non-replicating RV vaccine candidate with high immunogenicity and cross-protection, a peptide containing a VP8* neutralizing epitope of a P[8] RV strain and a 150 amino-acid long consensus sequence derived from a wide range of human P[8] strains was constructed [54], expressed, and purified from recombinant *E. coli* [55]. This fusion peptide was recognized by a large number of RV-infected patients’ sera, and its immunogenicity was proven in pilot mouse experiments. The authors are aware that testing for protective efficacy will be required and will be the subject of future work [55]. In this context, it should be remembered that an rRV with VP6 mutations has been created which does not produce infectious viral progeny while expressing viral proteins in immunogenic concentrations [33].

High titers of maternal, RV-specific antibodies may be a factor contributing to a decrease in vaccine efficacy in low- and middle-income countries. This possibility was investigated in >500 mother–infant pairs as part of an RV3-BB vaccine trial in Malawi [56]. Maternal anti-RV IgG, but not IgA levels, were correlated with reduced takes after several doses of the neonatal vaccine, but not at the end of the study, leading to the conclusion that the RV3-BB 3-dose neonatal vaccine schedule may have the potential to protect against severe RV disease [56].

Chimeric human–mouse recombinant rotaviruses [18,19] provide a new strategy for studying human rotavirus-specific immunity and can be used to investigate factors causing variability in rotavirus vaccine efficacy. The genome of the human RV vaccine strain RIX4414 (Rotarix^®^) was used with the aim of generating an authentic live attenuated rRV [15] with biological characteristics very similar to those of the parental virus 89-12 (G1P[8]) [16]; this will enable the identification of attenuation mutations and the rational design of NG RVA vaccines.

The use of mRNA-based therapeutics and vaccines [57,58] has led to the recent successes of the mRNA-1273 SARS-CoV-2 [59] and the BNT162b2 mRNA COVID-19 [60] vaccines. Lu et al. [61] constructed a recombinant plasmid containing a wildtype VP7 (G1) gene as insert from which RV RNA was transcribed and capped in vitro, followed by purification and enclosure into lipid nanoparticles (LNPs). In mice, the VP7-LNP elicited RV- specific antibodies and activated T cells. Testing for protective efficacy is underway [61]. Recently, mRNA-based RV-vaccine candidates were shown to exert partial protection in the gn piglet model of RV Wa (G1P[8]) infection [62].

In order to understand the dynamics of systemic and mucosal immune responses early in life, studies on T cells in mice have been initiated. It was shown that early life T cells have a low capacity to generate long-term memory compared to those of adult mice. Similarly, T cells in human neonates are immature and evolve only with age. At present, it is not clear how T cell diversification in early life affects the development of clinical symptoms after infection [63].

As co-guest editors, we are acutely aware that the >20 contributions to RV research collated in this SI of RVs and RV vaccines represent only a relatively small sector of a multitude of ongoing original work, implying that RV research still faces large gaps in regard to knowledge and understanding. However, with the discovery and application of new biological tools, including refined reverse genetics systems, many aspects of rotavirus biology will become amenable to study. These include:

- Developing rotavirus vaccine candidates that are more immunogenic and grow to high titers;
- Probing the usefulness of rotaviruses as vaccine vector systems through their capacity to incorporate and express heterologous sequences;
- Understanding the molecular basis for partial gene duplications, and their selection, in RVA isolates in vivo and in vitro;
- Revealing the contributions of cellular components to the RV–host relationship and pathogenesis;
- Expanding rudimentary knowledge of the biology, epidemiology, and pathogenesis of the non-species A RVs;
- Establishing the basis for the difference in efficiency of RV vaccines in different parts of the world, an effort that is vital to improving protection in low socio-economic regions where children are most at risk due to rotavirus disease.

## Figures and Tables

**Table 1:** Rescue of species A rotaviruses by reverse genetics.

Virus	References
Simian SA11 G3P[2]	[8,9]
Human rotavirus Ku G1P[8]	[11]
Human rotavirus Odelia G4P[8]	[12]
Human rotavirus CDC-9 G1P[8]	[13]
Human rotavirus G2P[4]	[14]
Human rotavirus RIX4414 G1P[8]	[15,16]
Simian rotavirus RRV G3P[3]	[17]
Murine rotavirus EW/ETD G3P[17]	[17,18,19]
Avian rotavirus PO-13 G18P[17]	[20]
Porcine rotavirus OSU G5P[7]	[21]
Bovine RV RF G6P[1]	[22]
Lanzhou lamb rotavirus LLR G10P[15]	[23]
Species A rotavirus reassortants (selection)	[17,19,23,24,25,26,27]
